# Deadly Marburg virus in Ghana, 2022 amidst monkeypox and COVID-19 pandemic: A distressing concern for public health

**DOI:** 10.1097/JS9.0000000000000161

**Published:** 2023-03-03

**Authors:** Aroma Naeem, Shehroze Tabassum, Syed M.H. Zaidi, Qasim Mehmood, Farhan Naeem, Saima Gill, Andrew A. Wireko

**Affiliations:** a King Edward Medical University, Lahore, Pakistan; b Sumy State University, Sumy, Ukraine

*Dear Editor*,

Marburg virus (MARV) is a rare yet deadly pathogen, an RNA virus belonging to the order: Mononegavirales, family: Filoviridae, genus: *Marburgvirus.* It is closely related to the Ebola virus. *Marburg marburgvirus*, only species causing a zoonotic disease called Marburg virus disease (MVD) in humans, manifests as skin rash, nausea, vomiting, malaise, hemorrhage, and fever^1^. MVD is a serious illness with case fatality rates reported to be as high as 80–90%^1^. MARV host reservoir is an African fruit bat *Rousettus aegyptiacus*. Since African fruit bats reside in caves and are extensively distributed across Africa; these might have infected humans, although the exact mechanism of transmission from fruit bats to humans is still unknown^2^. The incubation period of MVD in Ghana is 5–10 days with sudden onset of fever, malaise, headache, chills, and myalgia; however, the disease is progressive and can cause pancreatitis, liver failure, weight loss, massive hemorrhage, shock, delirium, and multiorgan failure^2^. It can spread to humans via direct contact with the body fluids of infected persons and fomites^3^. Early confirmation of MVD is made by immunoglobulin M capture enzyme-linked immunosorbent assay, PCR, and antigen-capture enzyme-linked immunosorbent assay. On 17 July 2022, a recent outbreak of MVD was declared by Ghana when they confirmed first two MVD cases from a region Ashanti^4^. Both cases tested positive for MARV on reverse-transcriptase PCR. The results were confirmed by the Noguchi Memorial Institute of Medical Research (NMIMR) on 14 July 2022^5^. MVD is reported to be the second zoonotic disease, West Africa has detected^3^.

MVD is a fatal infection with a significant potential to become an epidemic^5^. Ever since the emergence of two significant epidemics of MARV in 1967, outbreaks and individual cases of MARV have been reported from Kenya, Uganda, South Africa, and the Demographic Republic of Congo (DRC)^6^. In 2008, two tourists who entered Rousettus from Uganda caves were reported to have separate incidents of MARV^7^. First ever outbreak of MVD in West Africa was declared to be in Guinea in 2021 when a case of MAVR was confirmed by PCR on 3 August 2021, and was declared dead on 4 August 2021^8^. Later in 2022, two confirmed cases of MVD in Ghana were discovered in a region called Ashanti. One of these cases was a 26-year-old man who reported to the hospital on June 26, and was declared dead by June 27. The other case was a 51-year-old male who visited the hospital on June 28 and was reported dead on the same day. Both confirmed cases had fever, nausea, vomiting, and diarrhea, and were treated in the same hospital^3^. Both patients belonged to a community with a forest environment. This addresses the need to exclude other causes of viral hemorrhagic fevers such as Ebola, typhoid fever, rickettsia, malaria, plague, and leptospirosis^5^. After confirmation of these two cases, a total of 108 residents of Africa were recognized as contacts of these two patients. There were 48 contacts from Savannah, 50 from Ashanti, and 10 from the Western region of Africa, who were self-quarantined with monitoring daily for 21 days. Out of these, only one reported symptoms but the blood sample collected from the patient having symptoms turned out to be negative at confirmation by NMIMR. All other persons remained asymptomatic throughout the follow-up period^5^. On 27 July 2022, two more cases of were reported in Ghana, and the total cases rose to four, with three deaths^2^. Little knowledge about the exact mode of transmission of the disease, along with the death toll in the recent outbreak of MVD in Ghana, highlights the threat this virus poses. Table 1 2,9–12 shows the distribution of MVD in different countries.

**Table 1:** Distribution of Marburg virus disease

Country	Year	Total number of cases
South Africa^9^	1975	3
Kenya^10^	1980	2
	1987	1
Angola^9^	2004–2005	374
Democratic Republic of Congo^11^	1998–2000	154
Uganda^12^	2007	4
	2008	2
	2012	15
	2014	1
	2017	3
Ghana^2^	2022	4

Due to ongoing COVID-19 pandemic and overlapping features between COVID-19 and MVD, it becomes difficult for a physician to accurately differentiate COVID-19 and MVD. Table 2 1,2,13,14 provides a comprehensive comparison between COVID and MVD.

**Table 2:** Comparison between COVID and MVD

Features	MVD	COVID-19
Agent	Marburg virus (MARV)	SARS-CoV-2
Incubation period (days)	5–10	4–14 (average 10)
Transmission	Through fruit bats Contact with infected person Through body fluids of infected persons	Respiratory droplets
Clinical course	Progressive and severe	Mild to critical
Signs and symptoms	Fever Malaise Headache Chills Myalgia Weight loss	Fever, chills, malaise, body weakness Sore throat, shortness of breath, cough Headache Anosmia (loss of smell), Agusia (loss of taste) Running nose Nausea and vomiting Diarrhea
Disease complications	Pancreatitis Liver failure Massive hemorrhage Shock Delirium Multiorgan damage	Shock Respiratory failure Multiorgan dysfunction

COVID-19, coronavirus disease 2019; MVD, Marburg virus disease; SARS-CoV-2, severe acute respiratory syndrome coronavirus 2.

No antiviral has been accepted as the appropriate treatment for MVD, neither is any approved vaccine for it^5^. Missing close contact, such as people living in the same house and healthcare workers, accelerates disease transmission. MVD, with a case fatality ratio of 24–88%, presents a serious public health threat to the people of Ghana^5^. Poor and underdeveloped healthcare system of Ghana has contributed to several outbreaks in previous years like Ebola, polio, yellow fever, and COVID-19. Lack of access to basic healthcare facilities, inadequate infrastructure, poor transport system and scarce ambulance services are the key hurdles in achieving disease control in the countries of West Africa, especially Ghana. Furthermore, the disease surveillance in Ghana is not so robust, and many healthcare centers are already overwhelmed, leading to poor response to MVD. People living in affected regions are not well-informed about the disease, thus putting an extra burden on the emergency response teams. Failure to combat this plague can pose serious risk of spread to the neighboring countries. The spread of this virus is high at a national level and moderate at a regional level as assessed by the WHO^5^. Movement of people across the border can cause further spread of this disease in West African regions like Sierra Leone and Liberia. Moreover, authorities are also concerned about the use of this virus as a bioweapon, as it has the potential to be aerosolized^15^.

World is already afflicted with multiple outbreaks^16^–^18^, this outbreak can be a potential threat to public health safety. Interrupting direct human-to-human and human-to-bat transmission should be the major objective of managing an MVD outbreak. People should avoid visiting caves where fruit bats are extensively distributed, or should adopt adequate protective measures before visiting. Travel outside of Africa has been a major factor in the transmission of MARV^19^,^20^. Therefore, a quick diagnosis is necessary to spot the sick before they spread the disease to other nations. Proper hand hygiene should be practiced while wearing personal protective equipment (PPE), such as gowns, gloves, masks, face shields, and goggles, to prevent contact with blood and bodily fluids. Patients and suspects must be housed in a separate room with attached bathroom to minimize physical contact with others. The safe disposal of virus-infected bodies is also recommended. Wildlife should be handled by using appropriate PPE. Reinforcing healthy eating practices is also vital. Creating an infection prevention and control activities task force in the health zone to ensure the implementation of infection prevention and control measures can lower transmission risk. Multisectoral action committee should be established and a proper plan should be devised^7^. Campaigns, including outreach programs in schools, hospitals, markets, and town hall meetings should be launched to spread awareness. Awareness should be raised through both print and electronic media. Medical professionals and researchers must be aware of the presence of MARV, together with the advent of infectious variants, such as the delta variant of SARS-CoV-2. Providing healthcare workers with adequate PPE is also essential. Health facilities should have the necessary capacity and WASH (water, sanitation, and hygiene) systems in place^5^. The government must devise strategies to prioritize funding for research, vaccine development and antiviral therapy. There should also be thorough investigations that can lead to a better understanding about the genetic makeup and discovery of hosts of MARV. Furthermore, building cross-border partnerships with global organizations, such as One Health, Food and Agriculture Organization (FOA), Office International des Epizooties (OIE), and WHO is essential to limit this deadly outbreak^21^.

## Provenance and peer review

Not commissioned, externally peer reviewed.

## Ethical approval

Not applicable.

## Sources of funding

None.

## Authors’ contribution

Conceptualization and editing: A.N. Writing: A.N., S.T., S.M.H.Z., Q.M., F.N., S.G., A.A.W. Review with critical comments: A.N. and S.T.

## Conflicts of interest disclosure

The authors declare that they have no financial conflict of interest with regard to the content of this report.

## Research registration unique identifying number (UIN)

Not applicable.

## Guarantor

All authors.
